# Supplementary material for: Broadband near-infrared emission in silicon waveguides
Source: Nat Commun. 2024 May 31;15:4639. doi: 10.1038/s41467-024-48772-6 (PMC11143322; doi:10.1038/s41467-024-48772-6)
Supplement: Supplementary file 1 — Supplementary Information [file 41467_2024_48772_MOESM1_ESM.pdf]

Supplementary Information: Broadband Near-Infrared Emission in Silicon Waveguides

Marcel W. Pruessner[1*†], Nathan F. Tyndall[1†], Jacob B. Khurgin[2], William S. Rabinovich[1], Peter G. Goetz[1] and Todd H. Stievater[1†]

[1*]Naval Research Laboratory, Washington D.C., USA.

[2]Dept. Electrical and Computer Engineering, Johns Hopkins University, Baltimore, MD, USA.

†These authors contributed equally to this work.

Abstract

This document contains supplementary information for: *Broadband Near-Infrared Emission in Silicon Waveguides.*

Keywords: PIC, LED, Hot carrier, waveguide, silicon photonics, p-i-n diode, integrated photonics, near-infrared

1 Emitter Design and Fabrication

The silicon p-i-n waveguide emitters were fabricated in a state-of-the-art 300-mm foundry integrated photonics process (AIM Photonics) using the available silicon active 220 nm thick device layer. Our design is fully compatible with the existing Multi-Project Wafer (MPW) foundry process and required no changes to the design rules or layer structures. Consequently, we expect that the emitters can be readily incorporated in future MPW runs. The rib waveguides, etched 110 nm deep, are 550 nm wide, and the full-etched waveguides are 480 nm wide as shown in Fig. 1. Note that the intrinsic region is confined entirely to the waveguide rib for the for the (nominally) d_{in} = 400 nm device (Fig. 1(a)) whereas the intrinsic region overlaps both the rib and slab regions for the d_{in} = 800 nm device (Fig. 1(b)). We calculated the p-i-n breakdown

voltage assuming a breakdown field [1] of 370 kV/cm and find good agreement with our measured reverse-bias breakdown (Fig. 2).

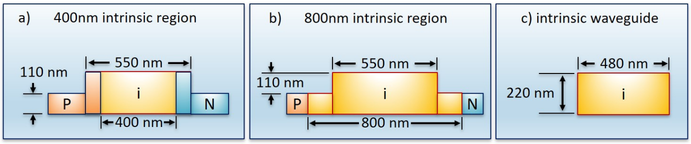

Supplementary Fig. 1 Waveguide cross-sections: a) d_{in} = 400 nm rib waveguide, b) d_{in} = 800 nm rib waveguide, c) full-etch input/output waveguides. The rib waveguides in (a) and (b) are 550 nm wide, and the full-etch waveguide in (c) is 480 nm wide. All waveguides have an SiO_2 cladding.

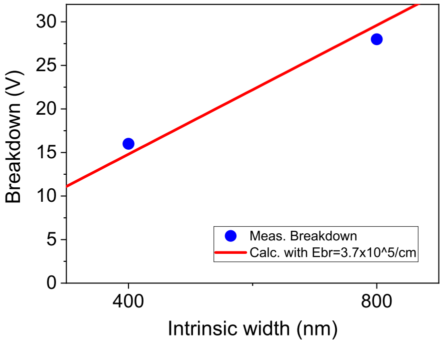

Supplementary Fig. 2 Measured and calculated breakdown voltage vs. intrinsic width assuming a breakdown field of 370 kV/cm.

2 P-I-N Burn-In and Effect of Doping

As can be expected at breakdown, small changes in the reverse-bias voltage result in large changes in the breakdown current. If care is not taken to limit the reverse-bias current, then significant device heating and potential dopant migration can occur. In Fig. 3 we show I-V measurements on a d_{in} = 800 nm device. The initial I-V shows a sharp breakdown at -28.3 V. After operation at large reverse-bias (-32 V) we observe a shift in the breakdown voltage indicating permanent changes to the p-i-n region likely due to dopant migration. Further operation at -32 V reverse bias results in an additional shift in the measured breakdown voltage and additional changes to the p-i-n behavior. Similar burn-in behavior is observed for the d_{in} = 400 nm device, but at smaller reverse-bias voltages. Limiting the breakdown current is key to enabling reproducible device behavior.

In our emission measurements we performed an initial burn-in step in which we carefully applied a reverse-bias to achieve breakdown. We set a current

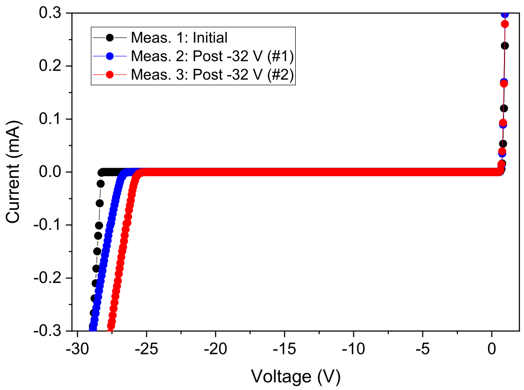

Supplementary Fig. 3 Measured change in I-V characteristics (d_{in} = 800 nm) after high applied electric field operation. The initial breakdown voltage (black curve) is shifted to smaller reverse-bias voltage (blue and red curves) indicating permanent changes to the intrinsic region due to e.g. dopant or defect migration for prolonged large electric fields.

limit of a few mA on our source and operated the p-i-n while adjusting the voltage to achieve a target current. Generally, the bias required to achieve a target reverse current stabilized after a few minutes of continuous operation. Subsequent measurements were highly-reproducible after this initial burn-in (see 11 Long-Term Emission section). Future devices can include an on-chip current source in order to achieve the required current limiting to prevent damage to the p-i-n due to e. g. thermal runaway at large currents.

We also investigated emitters with different doping levels. Although the precise doping concentrations are not shared by the foundry, we found only small differences in the reverse-bias breakdown voltage and no change in the forward-bias turn-on voltage (Fig. 4). Imaging of the p-i-n junctions showed that device emission under forward- and reverse-bias occurred for all devices and doping levels investigated.

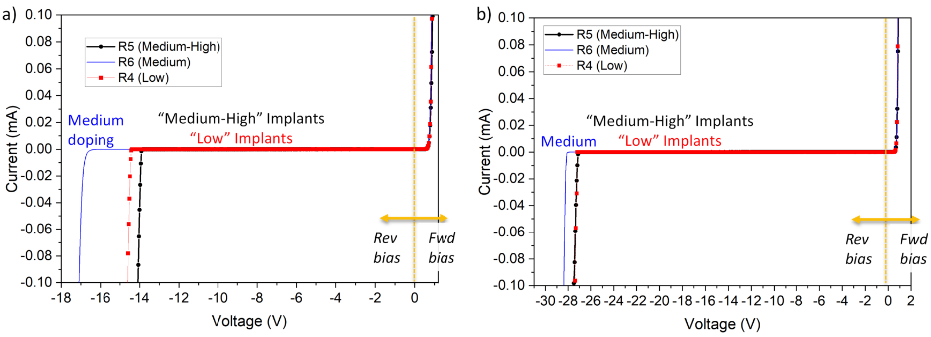

Supplementary Fig. 4 Measured I-V characteristics for devices with different doping levels: a) 400 nm intrinsic region, b) 800 nm intrinsic region.

3 Rib Waveguide Properties

Fig. 5 shows the calculated effective index of the waveguide modes in the rib emission region, as well as the slab mode of the silicon. All of the calculated results in this section are found using Comsol Multiphysics Electromagnetic Waves. At the shortest wavelengths, the TM_{00}, TE_{10}, and TE_{00} modes can all be populated by hot-carrier emission. Near 1000 nm the TE_{10} and TM_{00} modes are mixed. As the wavelength increases, the less-confined mode (which becomes predominantly TM_{00} in nature) is degenerate with the TE slab mode at approximately 1080 nm. This degeneracy leads to increasing loss for TM-polarized light as the wavelength increases (see inset of Fig. 5a) [2]. The TE_{10} mode continues until approximately 1450 nm, at which point its effective index is also degenerate with that of the silicon slab and couples out of the waveguide. Since this mode is odd, it is filtered out by the use of single-mode fiber and is not observed at our detector. Though a ridge waveguide (no slab) has different modal dispersion than a rib, the devices measured here are cleaved such that emission in the rib is coupled to the collection fiber with little to no propagation in the silicon ridge waveguide. Any propagation in the ridge waveguide would not be expected to add to any polarization-dependent loss due to that of the rib.

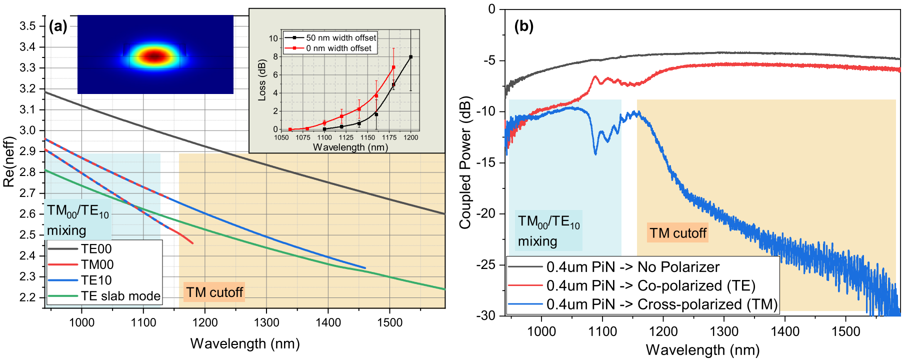

Supplementary Fig. 5 **a)** Calculated n_{eff} vs. wavelength for the supported modes of the silicon rib emitter as well as the TE silicon slab mode. *Left Inset*: The axial component of the Poynting vector for the TE_{00} mode at 1310 nm. *Right Inset*: The calculated loss of the TM_{00} mode due to slab coupling, for two rib widths (error bars are calculational loss uncertainty due to a finite-sized model. **b)** Measured emission from a d_{in} = 400 nm fiber-coupled device through an inline U-bench, normalized by the emission measured without the U-bench.

To corroborate the simulations, we measured the polarization of the emitted light as a function of wavelength. In Fig 5(b), we plot the optical power coupled into the spectrometer from the collection fiber through a U-bench containing no polarizer, or a linear polarizer. Each curve is normalized to the spectrum of the source emission, collected via PM fiber and directed into the spectrometer, bypassing the U-bench. Consequently, the black curve represents the U-bench transmission, with no polarizer, as a function of wavelength.

Then, we inserted a linear polarizer parallel to the PM fiber slow axis, to measure the light emitted into the TE mode(s) of the waveguide (shown in red). Finally, we rotated the polarizer perpendicular to the fiber slow axis, to measure the light emitted into the TM mode of the waveguide and the fast axis of the fiber (shown in blue). We observe two prominent features that correspond to simulated emitter modes. First, the TM_{00}/TE_{10} mode beating occurs from 1050 nm to 1100 nm, consistent with our rib waveguide model. Second, the TM_{00} mode cuts off near 1150 nm, also consistent with our model. Small fabrication-based uncertainties in the rib etch depth and width will shift the measured mode cutoff wavelength, as shown in the inset of Fig. 5(a). Finally, although outside the scope of the present work, long wavelength operation into the mid-IR (useful for spectroscopic applications) requires careful design of the waveguide to prevent excessive propagation loss due to low mode confinement effects such as bend loss or substrate loss.

Together, these simulations and measurements show that for wavelengths larger than approximately 1150 nm the emission is predominantly in the TE modes, due to slab coupling of the TM_{00} at these wavelengths. For wavelengths larger than approximately 1450 nm, the TE_{10} mode is coupled to the slab so the device operates only in the TE_{00} mode. At wavelengths shorter than approximately 1100 nm, the emission populates the TM_{00}, TE_{10}, and the TE_{00} modes.

To calculate the waveguide absorptivity, we require the material indices and group indices as well as the modal effective index and group index. These calculated parameters are shown in Fig. 6. The SiO_2 material index is based on ellipsometry measurements provided by the foundry, and the silicon material index is based on measurements from Green *et al.* [3].

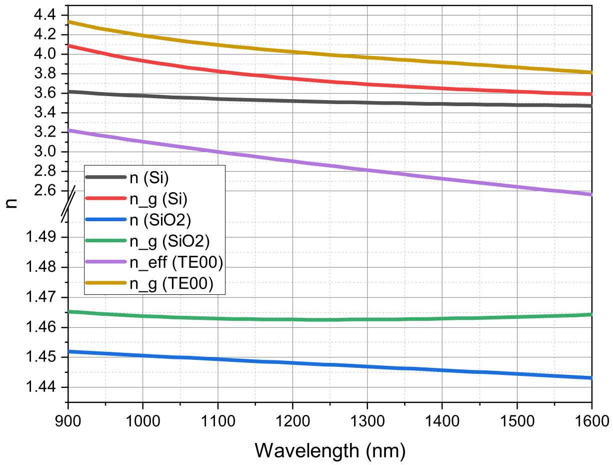

Supplementary Fig. 6 Material index and group index for Si and SiO_2, as well as the effective index and group index for the TE_{00} mode.

The modal overlap (Γ) between the intrinsic region and the TE_{00} mode determines how much of the free-carrier absorption contributes to the waveguide emissivity. This overlap is calculated for the 400 nm wide and 800 nm wide intrinsic widths and is shown in Fig. 7. These calculations indicate that the overlap factor for these two designs differ by 15% or less, depending on the wavelength. Thus, any observed differences in the emission spectrum larger than that for devices with similar currents likely arise from differences in the details of the propagation losses between the emitter and the facet, or the coupling between the facet and the collection fiber.

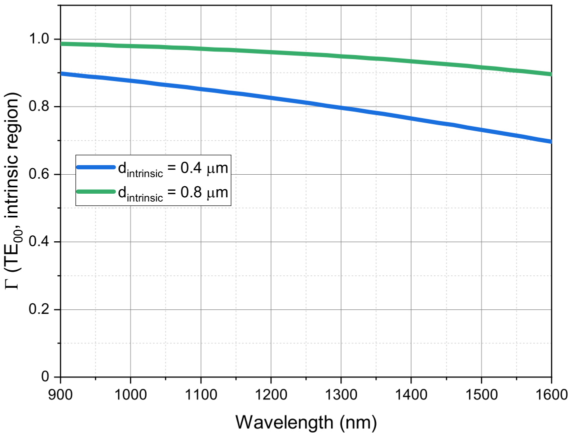

Supplementary Fig. 7 The calculated modal overlap, Γ, between the TE_{00} mode and the intrinsic region.

The waveguide geometry can support a TE_{00} mode to wavelengths beyond 3.0 μm. Fig. 8 shows the TE_{00} mode at a wavelength of 3.25 μm. The geometry is identical to that measured here, except that the rib is widened to 950 nm. The loss due to substrate leakage is found to be 2.2 dB/cm. As the hot carrier emission (HCE) also should extend out past 3.0 μm, this waveguide emitter along with passive silicon waveguide segments can be used as a broadband source and platform for spectroscopy from the silicon bandedge to the mid-wave IR.

4 Grating Emission

The surface-normal emission images were taken with an infrared camera mounted surface-normal to the device and waveguide grating. While the grating is convenient in enabling a simplified image capture of the p-i-n emission, gratings have a limited bandwidth. The grating emission (and collection) is given by the Bragg equation, and the emission angle is found using [4]

$$\sin\theta_m = (d_{grat} n_{eff} - \lambda_0)/d_{grat} n_{bkgrnd} \tag{1}$$

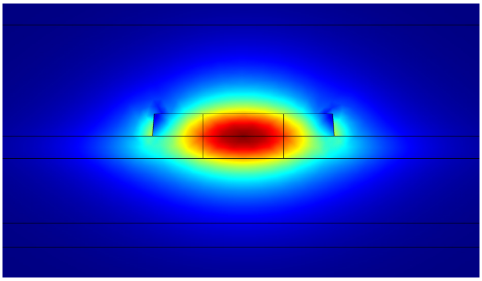

Supplementary Fig. 8 The calculated TE_{00} mode at a wavelength of 3.25 μm and a rib width of 950 nm.

where θ_m is the grating angle for grating order m, d_{grat} is the grating period, and λ_0 is the free-space wavelength, and n_{bkgrnd} is the background index into which the grating emits. When using a fixed lensed fiber the collected emission spectrum is determined primarily by the wavelength-dependent grating response. We have characterized the waveguide grating using a fiber array that enables input/output coupling between two on-chip gratings (GC-1 and GC-8) that are connected via a loop-back waveguide. The measured spectrum in Fig. 9 shows a peak grating transmission for the TE_{00} mode at 1507 nm with a 30 nm FWHM bandwidth. For this reason, the measured emission spectra are reported using devices with cleaved waveguide facets, which enable broadband coupling to single-mode optical fiber over hundreds of nanometers.

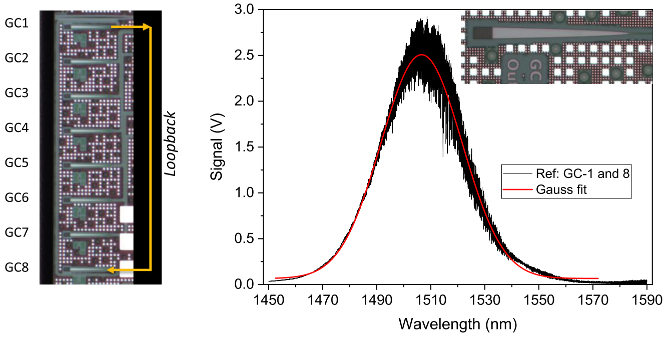

Supplementary Fig. 9 Measured grating response spectrum using a cleaved fiber array in which TE-polarized laser light is coupled intro grating 1 (GC-1) and collected from GC-8. A short waveguide segment connects GC-1 and GC-8 thereby enabling coupling alignment and characterization of the grating response.

5 Post-Processing: Waveguide Facets

The gratings exhibit a strong wavelength-dependent transmission that peaks near λ=1510 nm. In order to quantitatively measure the emission spectrum

over a broad wavelength range we cleaved the sample as close to the p-i-n emitter region as possible. The samples were laser scribed (Oxford Laser Micromachining Tool) and subsequently cleaved (Dynatex) in order to obtain waveguide facets with only very short waveguide segments relative to the p-i-n emitter region. Figure 10(a) shows an emitter chip after laser scribing with the expected cleave line indicated. We scribed and cleaved multiple samples. The measurements reported in the manuscript are from the device shown in Fig. 10(b) in which the cleaved facet results in a full-etched waveguide as opposed to a half-etched rib waveguide. The total waveguide length from the p-i-n emitter to the facet is 60 μm.

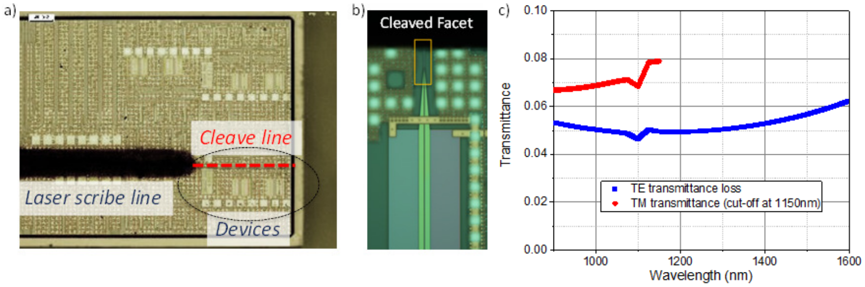

Supplementary Fig. 10 Post-processing: a) image of chip after laser scribing but prior to cleaving, b) image of cleaved waveguide facet to expose full-etched waveguide approximately 60 μm away from the p-i-n emitter region, c) simulated waveguide-fiber coupling (TE and TM).

To determine the optical power emitted by the waveguide junction, we normalized the measured spectra by the expected loss when coupling a lensed fiber to a silicon waveguide facet. This expected loss was determined via simulation (Fig. 10(c)) indicating broadband waveguide-fiber coupling over 900-1600 nm wavelengths for TE-polarization while the TM_{00}-mode is cutoff near 1100 nm as mentioned previously. Normalizing by the coupling efficiency provides the internal optical power in pW/nm as plotted in the main text.

The waveguide to fiber coupling simulation is performed separately for the TE_{00} and TM_{00} modes using the standard modal overlap integral for the electric fields. The electric field profile for the waveguide (rib) mode is found using Comsol Multiphysics (Electromagnetic Waves), and the fiber mode is described by a diffraction-limited (Gaussian) focused spot with a $1/e^2$ diameter of 2.0 μm at a wavelength of 1064 nm. The coupling also assumes a Fresnel reflection at the waveguide-air interface based on the wavelength-dependent effective index of the mode.

6 Intrinsic Region Width

We measure emission from devices with two different widths of the intrinsic region, d_{in}, of 800 nm and 400 nm. The emission should differ primarily in the

voltage required to break down the diode, since the breakdown field should be approximately equal for the two intrinsic widths. As shown in Fig. 11, this is what is observed. When biased at the same reverse-field, the emission would then only be expected to differ by the modal overlap, Γ, between the optical mode and the hot carriers. This modal overlap is shown in Fig. 7 above, and differs by less than 15% between the two designs. Since the observed spectra differ by more than that at shorter wavelengths, we believe that differences in the fiber-waveguide coupling and/or waveguide propagation losses primarily account for these differences.

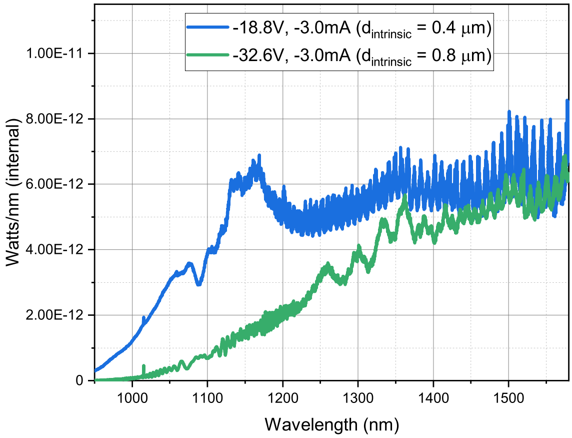

Supplementary Fig. 11 Measured reverse-biased emission spectra from two different devices with emitter regions with d_{in}=400 nm and d_{in}=800 nm.

7 Forward-Bias Emission

Our p-i-n emitter power was characterized under both forward-bias and reverse-bias conditions. Forward-biased silicon LEDs emit light near the band-edge (1100 nm wavelength) as has been reported by others [5–7]. However, silicon's indirect bandgap requires phonons for momentum conservation for recombination-based photon emission (Fig. 12(a)), which limits their emission efficiency. We integrated our measured forward-bias emission power spectral density and find a linear dependence on the current (Fig. 12(b)) as expected. Electroluminescence and forward-bias operation yielding bandedge emission in our p-i-n waveguide emitter is important for applications that do not require broad optical bandwidths. The broadband emission from reverse-bias is more generally applicable for photonic integrated device characterization and absorption spectroscopy and is the primary focus of this work.

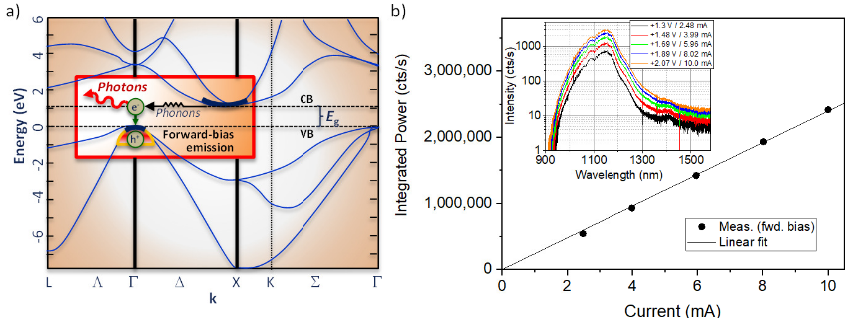

Supplementary Fig. 12 Forward-biased LED emission: **a)** Hot electrons in the conduction band recombine indirectly with holes in the valence band via phonons to emit primarily at the (interband) energy gap, **b)** measured forward bias (d_{in} = 400nm) integrated power vs. injected current; *Inset*: measured spectra showing emission at the bandgap wavelength near 1100 nm.

8 Reverse-Bias Emission

Broadband intraband emission from hot carriers in silicon can arise from a number of phenomena, including bremsstrahlung radiation, direct intraband transitions (between nearly degenerate conduction bands or valence bands), and/or indirect transitions within a single band. Previous observations of reverse-bias emission in surface-normal geometries in the visible have rejected bremsstrahlung emission as a primary mechanism due to its energy dependence [8–10], though they allow for some contribution at higher photon energies (>2 eV). Direct intraband transitions could occur near the X-point for conduction band electrons or near the Γ-point for valence holes. Since these transitions, however, are strictly forbidden at the X-point and the Γ-point, the amplitude of this radiation would be expected to increase with photon energy as the transition probability increases with the band separation. That trend is the opposite of what is observed in our measured reverse-bias spectra. This suggests indirect intraband processes as the most likely source for our reverse-biased infrared emission into the waveguide. Such a process would require scattering between the carriers and phonons (or perhaps material defects) to conserve momentum during the photon emission.

9 Joule Heating and Reliability

In both forward- and reverse-bias, Joule heating can contribute to a significant increase in the device (lattice) temperature. This heating can be accurately estimated using a three-dimensional finite-element model that assumes the silicon substrate provides a perfect heat sink for conductive heat flow from the intrinsic region through the bottom oxide. We use Comsol Multiphysics (Heat Transfer) to model this resistive heating. The simulation shows a temperature increase of the emitter of 250K for a resistive power of 100 mW, which approximately corresponds to the -5 mA reverse-bias (-21.3 V) case.

We investigated the thermal limit by operating a 400 nm wide intrinsic region emitter device at a large reverse bias current (-10 mA and -16.8 V). Imaging revealed highly-localized emission (”hotspots”) within the intrinsic region – in contrast to the uniform emission seen when operating at lower currents (see Fig. 1 in the manuscript). Continued operation at -10 mA resulted in device failure. Scaling our model to 170 mW applied electrical power results in a temperature increase of around 400 K suggesting a maximum operating condition *for this particular emitter device.* Furthermore, the burn-in process discussed in *Section 2* was not performed on this particular device which may have contributed to the thermal failure. Future modifications t o t he doping levels and profile m ay a lso a ffect br eakdown co nditions an d im prove device performance and reliability.

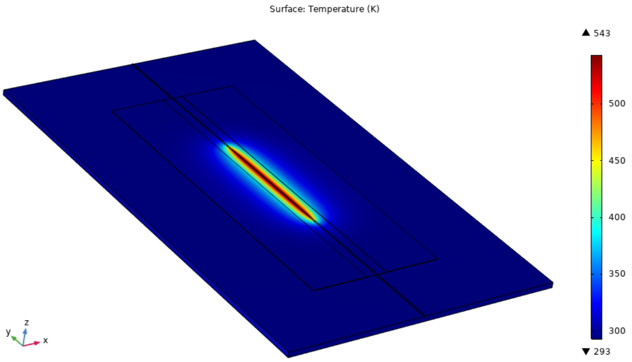

Supplementary Fig. 13 Finite-element simulation of Joule heating in the intrinsic region of the waveguide emitter for an applied electrical power of 100 mW.

10 Spectrometer Comparison

To show that this device can be used with compact, TE-cooled spectrometers, we directly compared the collected signal from a conventional benchtop spectrometer with a liquid-nitrogen cooled InGaAs detector (0.5-meter Czerny-Turner grating spectrometer, Princeton Instruments SP2558) to a compact spectrometer with a TE-cooled InGaAs detector (Wasatch Photonics NIR1-C). To perform this comparison, the collected emission in the PM fiber is simply attached to either the benchtop spectrometer or the handheld spectrometer, with no other change in experimental conditions. As shown in Fig. 14, the handheld spectrometer actually collects between 2× and 3× more signal (counts/s) than the benchtop detector, with no significant increase in noise. Notably, the handheld detector's exposure time was half that of the benchtop detector for these spectra. These spectra demonstrate that the hot-carrier emission is sufficiently bright for use with compact spectrometers for low SWaP sensor systems-on-a-chip.

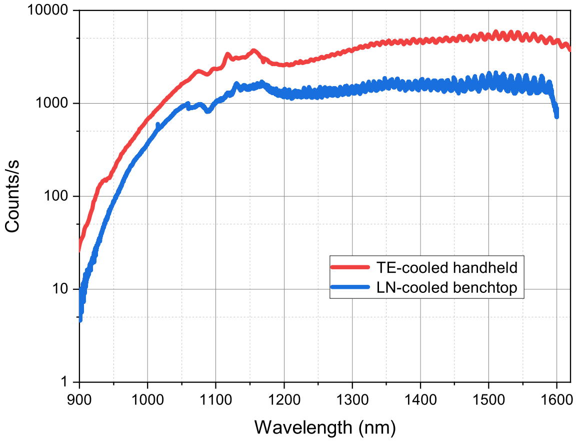

Supplementary Fig. 14 Measured raw emission spectra of the same device biased at the same reverse bias voltage (-17.6V) and reverse current (-2.0 mA) and collected into the same collection fiber, but sent to two different spectrometers.

11 Long-Term Emission

We operated the device continuously for over 4 days and measured the emission spectra as well as I-V curves periodically over that time. The I-V curves showed no discernible difference immediately after burn-in (t=0) compared to after 98.3 hours of continuous operation. Fig. 15 shows the measured spectra immediately after burn-in and then after 98.3 hours. We believe the primary contributor to the increased signal after continuous operation is improved alignment of the collection fiber.

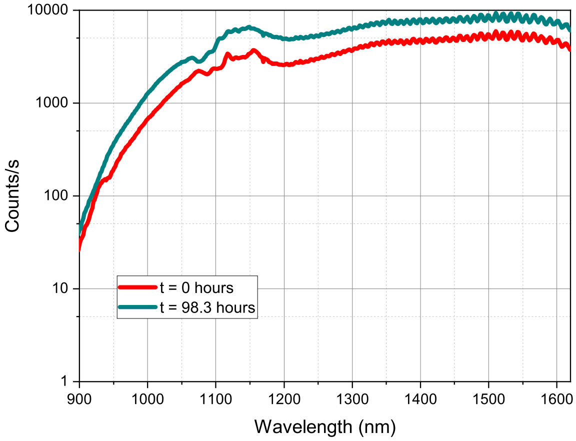

Supplementary Fig. 15 Measured raw emission spectra of the same device obtained after initial burn-in (t=0) and then after 98.3 hours of continuous operation. The reverse-bias varied by less than 0.03 V to maintain a -2.00 mA reverse current over this time.

12 Saturation Behavior

As discussed in the manuscript, more current can be injected into the diode to increase the emissivity of the device. The calculated absorptivity (αL) in this wavelength range for 5 mA is approximately 1×10^{-3}, suggesting 1000× more emitted power is possible with higher reverse currents. However, finite-element models of lattice heating due to diode resistance indicate that the local lattice temperature at the emitter rises by 250 K for electrical powers of 100 mW (corresponding to a reverse-bias current of 5 mA). Thus, thermal failure at the diode is likely to pose the ultimate limit to the achievable emitted power. Our simulations showed that heating is localized to the biased diode. However, thermal cross-talk due to lateral heat flow to adjacent devices may impact the overall scalability, and thermal isolation strategies using trenches and undercuts [11] may be used to further mitigate this concern.

Concerning optical power, saturation of the emissivity can already be observed at 4 mA for wavelengths near the silicon bandedge (900 nm to 1100 nm, as shown in Fig. 16), presumably since here the device has become effectively opaque (and is made only more so as the bandedge redshifts with Joule heating). At longer wavelengths (1200 nm to 1590 nm), no such saturation is observed at currents up to 5 mA, consistent with an emissivity limited by free-carrier absorption.

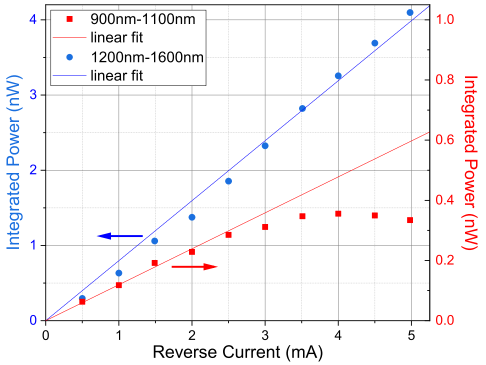

Supplementary Fig. 16 Measured power vs. reverse current for two wavelength ranges, as well as a linear fit to the reverse-bias currents < 2.5mA.

13 Source Signal-to-noise Ratio

Signal-to-noise ratio (SNR) is an important characteristic of a spectroscopic source, as it is a measurement of the quality of the source. Further, it can be used to estimate the limit of detection of a spectroscopic system. Here we extract the SNR for our broadband Si emitter as a function of wavelength, and use that to estimate our limit of detection for IPA, one of the analytes discussed in the main text.

Given the raw spectrum of the silicon emitter source (in counts, Fig. 17(a) below), coupled via lensed fiber into an LN-cooled InGaAs spectrometer, we extracted the signal to noise ratio (SNR) of the source. First, to remove low frequency features such as spectral shape and etaloning, we applied a high pass filter to the data. Then, we extracted the noise as a function of wavelength with a moving standard deviation, setting the bin size to 1.2 nm, which is equivalent to 10 pixels on our detector array. We converted this standard deviation from counts to counts/sqrt(Hz) using the exposure time (10 seconds) of the source measurement. The SNR plotted in Fig. 17(b) below is the result of dividing the raw signal (counts) by the noise (counts/sqrt(Hz)). It is important to note that this noise is likely not fundamental in nature (that is, it is not thermal background noise[12] or instrumentation noise) but is instead limited by measurement-based etaloning and other interferences. Single-chip sensors built with the source and the sensor on the same platform will reduce this noise significantly.

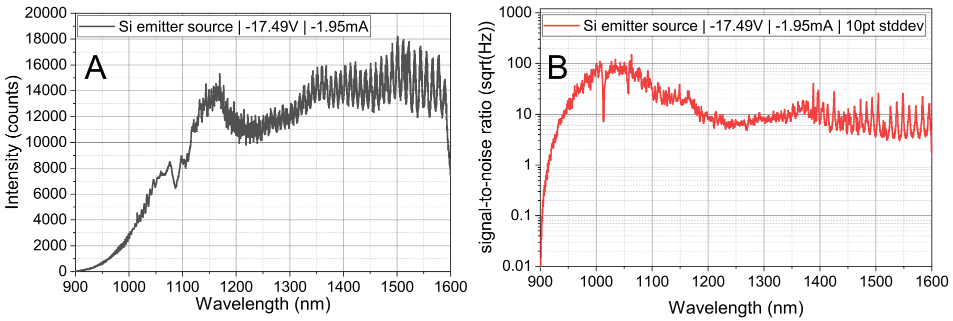

Supplementary Fig. 17 **(a)** Raw spectrum of Si emitter source collected via lensed fiber into LN-cooled InGaAs spectrometer. **(b)** Extracted source SNR as a function of wavelength.

With this measurement-limited SNR for our source, we can estimate a limit of detection for a relevant analyte based on our reported absorption spectroscopy data (Fig. 5 in the main text). For example, we clearly resolve an absorption peak at 1185 nm in the IPA absorption spectrum at an exposure time of 10 seconds. This yields a *measurement-specific* SNR of roughly 31. Based on this number, we would expect to resolve an IPA-specific peak at 1185 nm down to concentrations of 3% IPA, which is low enough for relevant applications such as analyzing mixtures of unknowns and monitoring analyte concentrations in liquid samples. This is an estimation of the detection limit for this spectroscopic architecture.

As with any source, we would expect the SNR to increase with increased emitter output, as well as with decreased loss between the source and the sensing spiral or with increased exposure time. As discussed in the main text, these proof-of-principle measurements required coupling the source light off-chip, then back on to a sensing chip that was exposed to the analyte. By

integrating the sensing spiral onto the same chip as the source, we would reduce the loss significantly and increase the SNR.

References

[1] Kano, K.: Semiconductor Devices. Prentice Hall, Upper Saddle River, New Jersey (1998)

[2] Webster, M.A., Pafchek, R.M., Mitchell, A., Koch, T.L.: Width dependence of inherent TM-mode lateral leakage loss in silicon-on-insulator ridge waveguides. IEEE Photon. Technol. Lett. **19**(6), 429–431 (2007). https://doi.org/10.1109/LPT.2007.891979

[3] Green, M.A., Keevers, M.J.: Optical properties of intrinsic silicon at 300 K. Progress in Photovoltaics: Research and Applications **3**(3), 189–192 (1995) https://onlinelibrary.wiley.com/doi/pdf/10.1002/pip.4670030303. https://doi.org/10.1002/pip.4670030303

[4] Van Acoloyen, K., Bogaerts, W., Jagerska, J., Le Thomas, N., Houdre, R., Baets, R.: Off-chip beam steering with a one-dimensional optical phased array on silicon-on-insulator. Optics Letters **34**(9), 1477–1479 (2009). https://doi.org/10.1364/OL.34.001477

[5] Green, M.A., Zhao, J., Wang, A., Reece, P.J., Gal, M.: Efficient silicon light-emitting diodes. Nature **412**(6849), 805–808 (2001)

[6] Zhao, J., Green, M.A., Wang, A.: High-efficiency optical emission, detection, and coupling using silicon diodes. Journal of Applied Physics **92**(6), 2977–2979 (2002) https://doi.org/10.1063/1.1503168. https://doi.org/10.1063/1.1503168

[7] Li, Z., Xue, J., de Cea, M., Kim, J., Nong, H., Chong, D., Lim, K.Y., Quek, E., Ram, R.J.: A sub-wavelength Si LED integrated in a CMOS platform. Nature Communications **14**, 882 (2023). https://doi.org/10.1038/s41467-023-36639-1

[8] Lacaita, A.L., Zappa, F., Bigliardi, S., Manfredi, M.: On the bremsstrahlung origin of hot-carrier-induced photons in silicon devices. IEEE Transactions on Electron Devices **40**(3), 577–582 (1993). https://doi.org/10.1109/16.199363

[9] Akil, N., Kerns, S.E., Kerns, D.V., Hoffmann, A., Charles, J.-P.: Photon generation by silicon diodes in avalanche breakdown. Applied Physics Letters **73**(7), 871–872 (1998) https://doi.org/10.1063/1.121971. https://doi.org/10.1063/1.121971

[10] El Ghazi, H., Jorio, A., Zorkani, I.: Analysis of silicon light emission under breakdown condition using an indirect intraband model. Optics Communications **281**(12), 3320–3323 (2008). https://doi.org/10.1016/j.optcom.2008.02.048

[11] van Niekerk, M., Deenadalayan, V., Rizzo, A., Leake, G., Coleman, D., Tison, C.C., Fanto, M.L., Bergman, K., Preble, S.: Wafer-scale-compatible substrate undercut for ultra-efficient soi thermal phase shifters. In: 2022 Conference on Lasers and Electro-Optics (CLEO), pp. 1–2 (2022)

[12] Boyd, R.W.: Photon bunching and the photon-noise-limited performance of infrared detectors. Infrared Physics **22**(3), 157–162 (1982). https://doi.org/10.1016/0020-0891(82)90034-3
